# Supplementary material for: Awareness of hepatitis B post-exposure prophylaxis among healthcare providers in Wakiso district, Central Uganda
Source: PLoS One. 2022 Jun 23;17(6):e0270181. doi: 10.1371/journal.pone.0270181 (PMC9223339; doi:10.1371/journal.pone.0270181)
Supplement: Attachment — Submitted filename: PLoS ONE-D-21-16912R1.docx [file pone.0270181.s004.docx]

Lines 70-72 – Change to “Chronic HBV infection remains one of the most serious of viral hepatitis, and is often associated with hepatocellular necrosis, inflammation, being cirrhosis and hepatocellular carcinoma the major complications”.

Lines 92-96 – Injection practices worldwide and especially in LMICs include multiple, available unsafe practices. Unsafe practices but are not limited to prevalent and high-risk practices, include: a) Reuses of injection equipment to administer injections to more than one person; b) accidental needle stick injuries in health-care workers; c) overuse of injection to health conditions where oral formulations are available; d) unsafe sharps waste management. Prevention of HBV infection in health-care setting include hand hygiene, safe handling and disposal of sharps and waste, safe cleaning of equipment, testing of donated blood, improved access to safe blood and training the health personnel. This manuscript is a opportunity to pass to the healthcare provides a clear message, regarding the awareness of hepatitis B among healthcare providers.

Lines 100-101 – Please correct to “…among HCPs (14,24)”

Line 106 – Please correct to “…post-exposure prophylaxis (PEP), resulting…”.

Lines 116-118 – Please correct to “HBV PEP include prevention of perinatal and early childhood HBV infection, persons who inject drugs, men who have sex with men, sex workers and healthcare providers.”

Line 133 – Please correct to “…seek PEP (35.36%).”

Line 135 – Please correct to “…HBV PEP among HCPs…”

Line 145 – Please correct to “Healthcare facilities (HCFs)…”

Line 146 – Please correct to “…which is designated as HC I, to HC II, III, IV, …”

Line 158 – Please correct to “…adequate knowledge on HBV PEP of 12.1% (40)…”

Line 176 – Please correct to “…management of HBV infection.”

Line 180 – Please correct to “…was awareness of HBV PEP options.”

Line 182 – Please correct to “…HBIG or HBV vaccine or both”

Line 200 – Please correct to “…(awareness of PEP options for HBV)…”

Line 202 – Please correct to “…of PEP options for HBV on…”

Line 233 – Please correct to table 2.

Lines 237,238,241,242,244,245,250,252,255 and tables 3 and 4 – Please switch Hepatitis B to HBV infection

Line 245 – Please correct to table 3

Line 257 – Please correct to table 4

Lines 258 and 259 – Please correct to table 4.

Lines 262, 264-266 – Please switch Hepatitis B to HBV infection.

Line 268 – Please correct to “…exposure to HBV infection (34).”

Line 270 – Please correct to “…HBV PEP.”

Lines 274,277,281,283,285,292,295,296,300,302,304,306-308,313,327,329 and 331 – Please switch hepatitis B to HBV

Lines 296,297,322 and 330 – Please use HCFs instead healthcare facilities.

Lines 309 and 310 – Please use HCPs instead healthcare providers.
