# Supplementary material for: Awareness of hepatitis B post-exposure prophylaxis among healthcare providers in Wakiso district, Central Uganda
Source: PLoS One. 2022 Jun 23;17(6):e0270181. doi: 10.1371/journal.pone.0270181 (PMC9223339; doi:10.1371/journal.pone.0270181)
Supplement: Attachment — Submitted filename: Response to Reviewers (1).docx [file pone.0270181.s006.docx]

**Response to Reviewers**

Dear Editor,

We would like to appreciate the critical review and comments provided by the reviewers. We have carefully addressed each comment as explained in the table below. We feel the quality of the manuscript has greatly improved.

| **Comment** | **Response** |
| --- | --- |
| **Journal Requirements:** |  |
| 1. Please review your reference list to ensure that it is complete and correct. If you have cited papers that have been retracted, please include the rationale for doing so in the manuscript text, or remove these references and replace them with relevant current references. Any changes to the reference list should be mentioned in the rebuttal letter that accompanies your revised manuscript. If you need to cite a retracted article, indicate the article’s retracted status in the References list and also include a citation and full reference for the retraction notice. | Thank you. We have carefully gone through the references, there are no changes to the previous version. |
| **Additional Editor Comments** |  |
| 1. There are numerous minor typos and inconsistencies to fix as the reviewer points out below. | Thank you. We carefully addressed each comment as explained below. |
| **Reviewer #1: Remain some minor additional comments** |  |
| Considering that healthcare providers (HCPs) is the same that healthcare workers (line 135), please uniform to HCPs | Revised |
| When a full name (e.g. continuous medical education – line 144) is used less than five times in the text there is no sense to use the acronym (e.g. CME) | Revised |
| Please uniform to hepatitis B PEP, and do not write the first letter of hepatitis with capital letter | Revised |
| Line 41… risk of hepatitis B virus… | Revised |
| Line 43… awareness of hepatitis B PEP… | Revised |
| Line 47… selected from healthcare facilities (HCPs)… | Revised |
| Line 77… burden of chronic HBV, … | Revised |
| Line 79… in the mortality due to human immunodeficiency virus (HIV)… | Revised |
| Line 81… Hepatitis B infection is… | Revised |
| Line 84… Hepatitis B infection accounts… | Revised |
| Line 89… Healthcare providers in SSA are… | Revised |
| Line 90… Healthcare providers have an up… | Revised |
| Line 93… especially in lower middle – income economies… | Revised |
| Table 1 In addition to services offered at healthcare centre IV… | Revised |
| … such as psychiatry, ear, nose and threat, ophthalmology… | Revised |
| General hospital | Revised |
| Regional referral hospital | Revised |
| National referral hospital | Revised |
| Line 112… precautions and PEP, resulting… | Revised |
| Line 120-121… Post-exposure prophylaxis is effective in the prevention | Revised |
| Line 122… Hepatitis B PEP… | Revised |
| Line 131… on PEP for hepatitis B, C and HIV 37 | Revised |
| Line 139… awareness of hepatitis B PEP | Revised |
| Line 141… hepatitis B PEP | Revised |
| Line 144… continuous medical education sessions | Revised |
| Lines 150-151… (10 hospitals, 15 health centres (HCs)… Healthcare facilities in Uganda… | Revised |
| Line 163… knowledge on hepatitis B PEP of 12,1% 43,… | Revised |
| Line 171… private for profit, private not for profit or public… | Revised |
| Line 185… hepatitis B PEP options… | Revised |
| Line 191… Healthcare facilities was considered… | Revised |
| Line 193… Healthcare providers were classified… | Revised |
| Line 205… (awareness of PEP options for hepatitis B)… | Revised |
| Line 206-207… of PEP for hepatitis B | Revised |
| Line 212… 95% confidence intervals are reported… | Revised |
| Line 224… Research and Ethics Committee. Administration… | Revised |
| Line 239 (Table 2) when acronyms are used in Tables (e.g. HCP and HCF), please use the full name as foot note at the end of the table or alternatively use the full name | Revised |
| Line 242… PEP for hepatitis B infection… | Revised |
| Line 246… PEP for hepatitis B. | Revised |
| Line 248… hepatitis B infection. | Revised |
| Line 250 (Table 3) It’s recommended to not use acronyms in tables (e.g. HBV, PEP, HBIG), however if it’s used please write the full name as foot note at the end of the | Revised. The full names have been added as foot notes at the end of the table (Line 253-254) |
| Line 257… PEP options for hepatitis B (AOR = 0.11, …) | Revised. |
| Lines 262-263 (Table 4) The same recommendations on the use of acronyms as for tables 1, 2 and 3 | Revised. The full names have been added as foot notes at the end of the table (Line 268-269) |
| Line 266… the awareness of hepatitis B PEP among… | Revised |
| Line 268… awareness of hepatitis B PEP among… | Revised |
| Line 269… that hepatitis B had PEP. | Revised |
| Line 270… of hepatitis B PEP may result… | Revised |
| Line 274… sensitive about hepatitis B PEP. | Revised |
| Line 279… options for hepatitis B 43 | Revised |
| Line 288… different hepatitis B PEP options… | Revised |
| Line 292… the hepatitis B PEP options… | Revised |
| Line 299 Healthcare providers in urban HCPs were more likely to be aware of hepatitis B PEP… | Revised |
| Lines 300, 304, 305, 307, 309, 310, and 315… hepatitis B PEP | Revised |
| Line 322… of hepatitis B PEP… | Revised |
| Line 329… hepatitis B PEP | Revised |
| Line 331… hepatitis B. Healthcare provider’s awareness of hepatitis B PEP… | Revised |
| Line 333, 334 and 335… hepatitis B PEP… | Revised |
