# Supplementary material for: Awareness of hepatitis B post-exposure prophylaxis among healthcare providers in Wakiso district, Central Uganda
Source: PLoS One. 2022 Jun 23;17(6):e0270181. doi: 10.1371/journal.pone.0270181 (PMC9223339; doi:10.1371/journal.pone.0270181)
Supplement: Attachment — Submitted filename: Response to reviewer comments PEP.docx [file pone.0270181.s005.docx]

**Awareness of Hepatitis B Post-Exposure Prophylaxis among Healthcare providers in Wakiso district, Central Uganda**

Response to comments/edits

| **Comment/suggestion** | **Response action** |
| --- | --- |
| Lines 70-72 – Change to “Chronic HBV infection remains one of the most serious of viral hepatitis, and is often associated with hepatocellular necrosis, inflammation, being cirrhosis and hepatocellular carcinoma the major complications”. | Thank you, has been revised accordingly |
| Lines 92-96 – Injection practices worldwide and especially in LMICs include multiple, available unsafe practices. Unsafe practices but are not limited to prevalent and high-risk practices, include: a) Reuses of injection equipment to administer injections to more than one person; b) accidental needle stick injuries in healthcare workers; c) overuse of injection to health conditions where oral formulations are available; d) unsafe sharps waste management. Prevention of HBV infection in healthcare settings includes hand hygiene, safe handling and disposal of sharps and waste, safe cleaning of equipment, testing of donated blood, improved access to safe blood, and training the health personnel. This manuscript is thus an opportunity to pass to the healthcare providers a clear message regarding awareness of hepatitis B of prevention. | Thank you, has been revised accordingly |
| Lines 100-101 – Please correct to “…among HCPs (14,24)” | Thank you, has been revised accordingly |
| Line 106 – Please correct to “…post-exposure prophylaxis (PEP), resulting…”. | Thank you, has been revised accordingly |
| Lines 116-118 – Please correct to “HBV PEP include prevention of perinatal and early childhood HBV infection, persons who inject drugs, men who have sex with men, sex workers and healthcare providers.” | Thank you, has been revised accordingly |
| Line 133 – Please correct to “…seek PEP (35.36%).” | Thank you, has been revised accordingly |
| Line 135 – Please correct to “…HBV PEP among HCPs…” | Thank you, has been revised accordingly |
| Line 145 – Please correct to “Healthcare facilities (HCFs)…” | Thank you, has been revised accordingly |
| Line 146 – Please correct to “…which is designated as HC I, to HC II, III, IV, …” | Thank you, has been revised accordingly |
| Line 158 – Please correct to “…adequate knowledge on HBV PEP of 12.1% (40)…” | Thank you, has been revised accordingly |
| Line 176 – Please correct to “…management of HBV infection.” | Thank you, has been revised accordingly |
| Line 180 – Please correct to “…was awareness of HBV PEP options.” | Thank you, has been revised accordingly |
| Line 182 – Please correct to “…HBIG or HBV vaccine or both” | Thank you, has been revised accordingly |
| Line 200 – Please correct to “…(awareness of PEP options for HBV)…” | Thank you, has been revised accordingly |
| Line 202 – Please correct to “…of PEP options for HBV on…” | Thank you, has been revised accordingly |
| Line 233 – Please correct to table 2. | Thank you, has been revised accordingly |
| Lines 237,238,241,242,244,245,250,252,255 and tables 3 and 4 – Please switch Hepatitis B to HBV infection | Thank you, has been revised accordingly |
| Line 245 – Please correct to table 3 | Thank you, has been revised accordingly |
| Line 257 – Please correct to table 4 | Thank you, has been revised accordingly |
| Lines 258 and 259 – Please correct to table 4. | Thank you, has been revised accordingly |
| Lines 262, 264-266 – Please switch Hepatitis B to HBV infection. | Thank you, has been revised accordingly |
| Line 268 – Please correct to “…exposure to HBV infection (34).” | Thank you, has been revised accordingly |
| Line 270 – Please correct to “…HBV PEP.” | Thank you, has been revised accordingly |
| Lines 274,277,281,283,285,292,295,296,300,302,304,306-308,313,327,329 and 331 – Please switch hepatitis B to HBV | Thank you, has been revised accordingly |
| Lines 296,297,322 and 330 – Please use HCFs instead healthcare facilities. | Thank you, has been revised accordingly |
| Lines 309 and 310 – Please use HCPs instead healthcare providers. | Thank you, has been revised accordingly |
